# Type III cryoglobulinemia associated with monoclonal gammopathy of uncertain significance and presenting with retiform purpura

**DOI:** 10.1016/j.jdcr.2023.11.002

**Published:** 2023-12-03

**Authors:** Michael J. Diaz, Vivian Liu, Mahtab Forouzandeh, Kiran Motaparthi

**Affiliations:** a University of Florida College of Medicine, Gainesville, Florida; b Department of Dermatology, University of Florida College of Medicine, Gainesville, Florida

**Keywords:** cryoglobulinemic vasculitis, mixed cryoglobulinemia, monoclonal gammopathy of uncertain significance, retiform purpura, type III

## Introduction

Cryoglobulinemia is a rare cause of complex purpura characterized by the presence of abnormal cryoglobulins in the serum that precipitate and dissolve with cold and warm temperatures, respectively.^1^ Three main variants of cryoglobulinemia exist: type I, in which the cryoprecipitate consists of monoclonal cryoglobulins^2^ and is associated with B cell lymphoproliferative disorders including monoclonal gammopathy of uncertain significance (MGUS); type II, which is caused by monoclonal and polyclonal cryoglobulins with rheumatoid factor (RF) activity; and type III, which involves polyclonal cryoglobulins with RF activity.^3^^,^^4^ Types II and III (mixed cryoglobulinemia) are most commonly associated with chronic infections, frequently hepatitis C virus (HCV); noninfectious causes include autoimmune diseases, particularly Sjögren syndrome; or systemic lupus erythematosus.^3^^,^^4^ Classically, type I cryoglobulinemia presents with retiform purpura^5^ and occlusive nonvasculitic vasculopathy, while mixed cryoglobulinemia presents with palpable purpura and small vessel vasculitis on histopathology. Here, we present a case of mixed (type III) cryoglobulinemia associated with MGUS and presenting with inflammatory retiform purpura.

## Case report

**Fig 1:**
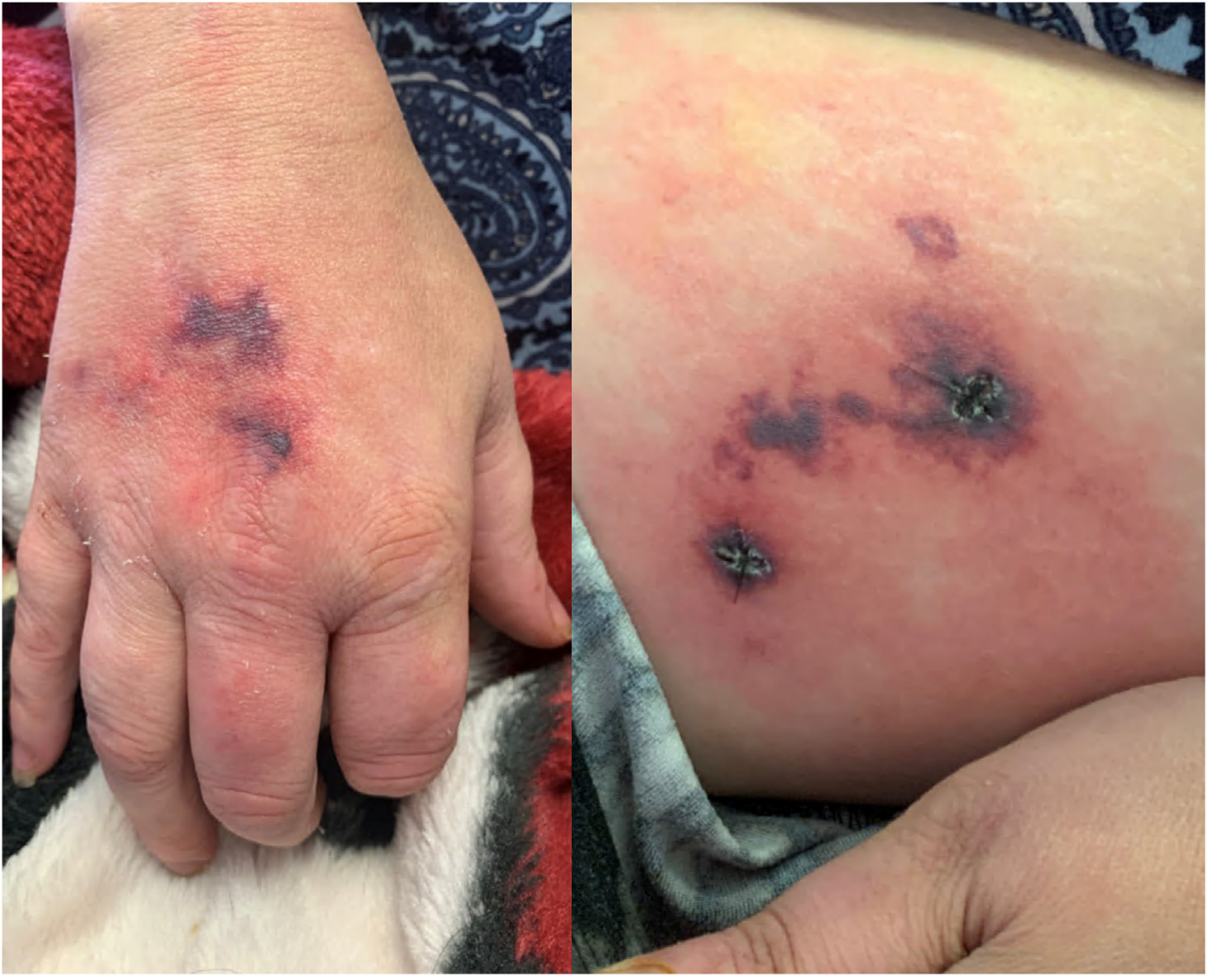
Inflammatory retiform purpura of the dorsal hand (*left*) and thigh (*right*).

A 47-year-old woman with a past medical history of plaque psoriasis, arthralgia, and neuropathy treated with gabapentin presented with a worsening ulcer on the calf. She had also experienced generalized weakness since hospitalization. Exam demonstrated a large ulcer with dusky gray border on the calf. On the hip and on the dorsal hands, there were retiform (stellate or branching) purpura with central necrosis but more prominent surrounding erythema (inflammatory retiform purpura, Fig 1). Punch biopsies (4 mm) demonstrated features of both an occlusive vasculopathy and a vasculitis affecting small vessels. Tissue culture was sterile, but blood cultures grew *Enterococcus faecalis*, *Klebsiella oxytoca*, and *Serratia marcescens*. At this time, the favored diagnosis was septic vasculitis given the clinical findings of a medium vessel vasculitis, the histopathologic findings demonstrating overlapping features of occlusive vasculopathy and vasculitis, and the clinical context of polymicrobial bacteremia. The patient was treated with intravenous cefepime and trimethoprim-sulfamethoxazole for a central line infection, with subsequent resolution of bacteremia but persistent cutaneous findings.

Two weeks later, additional inflammatory retiform purpura developed on the thigh, and the ulcer persisted despite several weeks of antimicrobial therapy. Repeat biopsy demonstrated similar overlapping features of occlusive vasculopathy and vasculitis. Urinalysis demonstrated hematuria (large) and proteinuria (100 mg/dL). Laboratory evaluation demonstrated positive qualitative serum cryoglobulin assay detected at 24 hours; serum free light chains demonstrated an elevated kappa:lambda ratio (2.02), and serum protein electrophoresis (SPEP) demonstrated an IgA kappa gammopathy. Quantitative analysis of the cryoprecipitate failed to identify detectable quantities of IgA, IgM, or IgG. RF assay was negative, and C3 and C4 levels were normal at this time. HCV serology was negative. Given these findings, a diagnosis of type I cryoglobulinemia was favored. However, subsequent bone marrow biopsy and flow cytometry of peripheral blood and bone marrow were unremarkable.

Due to the persistent clinical features and negative hematologic evaluation, SPEP and immunofixation, qualitative serum cryoglobulin and quantitative cryoprecipitate analysis, RF assay, and C3 and C4 were repeated; warming at 37 °C by submerging tubes in warm water was ensured following collection and during transport to the laboratory. A monoclonal IgA gammopathy with kappa restriction was identified once again. Serum cryoglobulins were identified again at 24 hours, and quantitative analysis of the cryoprecipitate demonstrated only polyclonal IgG, without any monoclonal proteins or IgA. RF was positive (18 IU/mL), and both C3 (69 mg/dL) and C4 (<8 mg/dL) levels were decreased. This repeat laboratory testing was consistent with type III cryoglobulinemia. Based on this diagnostic confirmation of mixed cryoglobulinemic vasculitis, laboratory evaluation was prompted for associated disorders. Repeat flow cytometry of peripheral blood was unremarkable again. HIV, hepatitis B virus, and repeat HCV serologies were negative. Antinuclear antibody, anti-Ro (anti-Sjogren syndrome A) and anti-La (anti-Sjogren syndrome B) serologic testing was negative.

Treatment was initiated with rituximab 1-g intravenous on day 0 and prednisone 40 mg daily to be tapered over 4 months. However, the patient experienced progressive weakness, arthralgia, new purpura, and gastrointestinal symptoms within days after rituximab infusion. These symptoms were thought to be due to precipitation of cryoglobulins with RF activity due to binding to rituximab. Therefore, the second infusion of rituximab on day 15 was deferred; plasmapheresis was initiated with prompt resolution of symptoms and healing of ulcers. The patient was also referred to nephrology given evidence of nephritis and to hematology for surveillance of B-cell lymphoproliferative disorders.

## Discussion

MGUS is defined by the presence of a non-IgM–type monoclonal protein in the serum with a level <3 g/dL, along with <10% clonal plasma cells in the bone marrow and absence of end-organ damage.^6^ Flavell et al reported a series of mixed cryoglobulinemia in association with MGUS and glomerulonephritis, including 1 case of type III cryoglobulinemic vasculitis.^7^ Cutaneous involvement was not described in this series; however, approximately 10% of cases of mixed cryoglobulinemia are regarded as idiopathic (essential).^8^ A B-cell lymphoproliferative disorder, including but not limited to MGUS, is almost always identified in association with type I cryoglobulinemia but can be variably identified in association with mixed cryoglobulinemia. Therefore, hematologic evaluation is necessary in patients with types II or III cryoglobulinemia without evidence of underlying infectious or autoimmune diseases. In either setting, patients with MGUS should be monitored for hematologic malignancies, which can present several years after the diagnosis of cryoglobulinemia. In patients with non-HCV mixed cryoglobulinemia, treatment should target the underlying condition and account for disease severity. For patients with no identifiable underlying disease, low-dose corticosteroids and colchicine may be used. First-line treatment options for those with severe disease include rituximab and corticosteroids or cyclophosphamide and corticosteroids. For patients with life-threatening disease, management consists of plasma exchange, pulse corticosteroids, and rituximab.^9^

**Fig 2:**
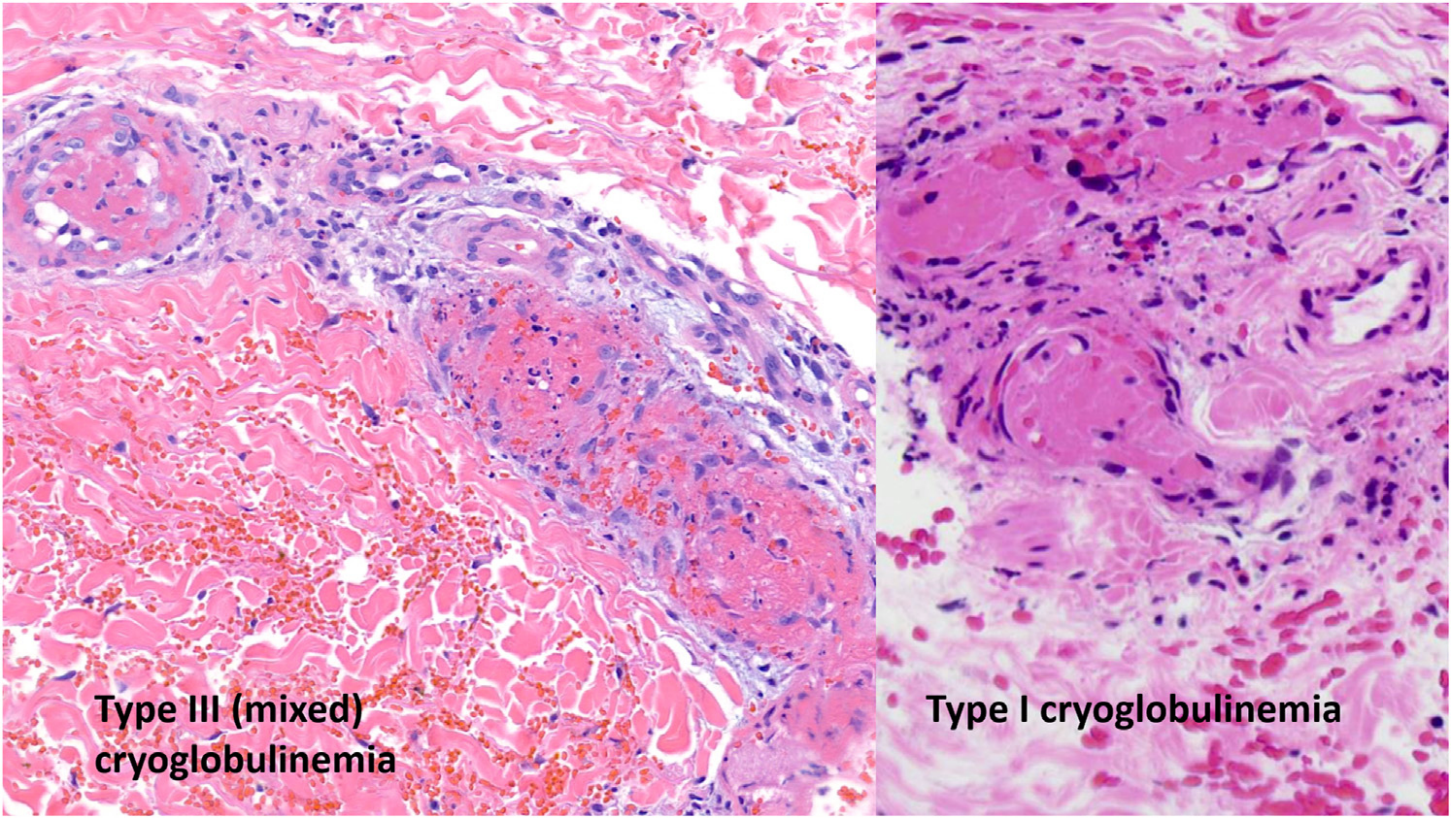
Skin biopsies of type III cryoglobulinemia (*left*, current case) and type I cryoglobulinemia (*right*). Both vasculopathy (occlusion by cryoprotein) and vasculitis are seen in both biopsies. Biopsy of type I cryoglobulinemia adapted from Bryan J, Skopis PK, Saikaly SK, Nguyen K, Motaparthi K. Comparing cases of type I cryoglobulinemia with histopathologic findings of vasculitis. JAAD Case Rep. 2022;23:160-161. Published 2022 Mar 2. https://doi.org/10.1016/j.jdcr.2022.02.014.

Interpretation of histopathology in the classification of cryoglobulinemia has traditionally dichotomized findings of vasculitis and occlusive vasculopathy in skin biopsies. However, overlapping features in the pathogenesis of type I and mixed cryoglobulinemia may also produce overlapping features seen in skin biopsies. Typically, type I cryoglobulinemia demonstrates occlusive vasculopathy: vascular occlusion by the circulating cryoglobulin. However, complement-mediated inflammation is sometimes identified in small vessel endothelia in type I cryoglobulinemia.^10^ Thus, both microvascular occlusion and vasculitic features can be observed in patients with type I cryoglobulinemia.^5^ In mixed cryoglobulinemia, circulating cryoglobulins in small vessels form immune complexes containing complement that result in fixation on endothelium and subsequent vasculitis. However, mixed histopathologic features of occlusive vasculopathy and vasculitis are not uncommon in mixed cryoglobulinemia.^10^ Immunoglobulins inconsistently form immune complexes, precipitate, and induce inflammation, which may explain the overlapping histopathologic features observed in type I and mixed cryoglobulinemia (Fig 2).^1^^,^^5^

For these reasons, the classification of cryoglobulinemia should not rely solely on histopathologic findings.^10^ Instead, differentiation of type I cryoglobulinemia from mixed cryoglobulinemia is based on clinical morphology, the presence of RF activity, serum complement levels, and the composition of the circulating cryoglobulins. Both type I and mixed cryoglobulinemia predominantly affect the extremities and acral sites. Type I cryoglobulinemia presents with noninflammatory retiform purpura owing to a mainly occlusive pathogenesis while mixed cryoglobulinemia presents with inflammatory retiform purpura and palpable purpura owing to vasculitis that affects both medium and small vessels, respectively. Livedo, Raynaud phenomenon, and ulcers are observed in both type I and mixed cryoglobulinemia.^1^ In type I cryoglobulinemia, cryoprecipitate analysis identifies a single monoclonal paraprotein also identified by SPEP. In contrast, in mixed cryoglobulinemia, the cryoprecipitate is composed of multiple proteins; SPEP may demonstrate monoclonal and polyclonal proteins (type II) or polyclonal proteins alone (type III). Importantly, blood must be warmed at collection, as this can affect the diagnostic yield during quantitative or qualitative testing. This is particularly impactful in mixed cryoglobulinemia, in which the pathogenic cryoprotein is often present in small or trace amounts. Table I provides a comparison of type I and mixed cryoglobulinemia based on consistently distinct findings.

**Table I:** Differentiation of type I cryoglobulinemia and mixed cryoglobulinemia

Feature	Type I cryoglobulinemia	Mixed cryoglobulinemia
Cryoprecipitate analysis	Monoclonal cryoglobulin (IgM, IgG, IgA, or Bence-Jones protein)^3^	Type II—monoclonal (IgM, IgG, or IgA) and polyclonal cryoglobulins (IgG); Type III - polyclonal cryoglobulins (IgM or IgG, IgA, or FLC)^3^
Complex purpura	Noninflammatory retiform purpura∗	Inflammatory retiform purpura† and palpable purpura
Rheumatoid factor	Negative	Positive
Serum complement levels (C3 and C4)	Normal	Decreased

*FLC*, Free light chains.

∗ Predominant necrosis surrounded by minor component of erythema.

† Minor component of necrosis surrounded by predominant erythema.

In summary, the presence of a monoclonal paraprotein (MGUS), retiform purpura, and overlapping histopathologic changes of occlusive vasculopathy and vasculitis may be observed in mixed cryoglobulinemia and confound distinction from type I cryoglobulinemia. Careful attention to clinical morphology and laboratory evaluation (quantitative cryoprecipitate analysis, RF activity, and complement levels) allow more reliable classification.

## Conflicts of interest

None disclosed.
